# Impact of infectious diseases on population health using incidence-based disability-adjusted life years (DALYs): results from the Burden of Communicable Diseases in Europe study, European Union and European Economic Area countries, 2009 to 2013

**DOI:** 10.2807/1560-7917.ES.2018.23.16.17-00454

**Published:** 2018-04-19

**Authors:** Alessandro Cassini, Edoardo Colzani, Alessandro Pini, Marie-Josee J Mangen, Dietrich Plass, Scott A McDonald, Guido Maringhini, Alies van Lier, Juanita A Haagsma, Arie H Havelaar, Piotr Kramarz, Mirjam E Kretzschmar, on behalf of the BCoDE consortium

**Affiliations:** 1 European Centre for Disease Prevention and Control (ECDC), Stockholm, Sweden; 2 Julius Center for Health Sciences and Primary Care, University Medical Center Utrecht, Utrecht, the Netherlands; 3 Centre for Infectious Disease Control, National Institute for Public Health and the Environment (RIVM), Bilthoven, the Netherlands; 4 Section Exposure Assessment and Environmental Health Indicators, German Environment Agency, Berlin, Germany; 5 Department of Public Health, Erasmus MC University Medical Center, Rotterdam, the Netherlands; 6 University of Florida, Gainesville, Florida, United States; 7 Mirjam E Kretzschmar (mirjam.kretzschmar@rivm.nl)

**Keywords:** burden of communicable diseases in Europe, disability-adjusted life years, infectious disease surveillance, public health policy, prioritisation in public health, influenza, HIV/AIDS, tuberculosis

## Abstract

The Burden of Communicable Diseases in Europe (BCoDE) study aimed to calculate disability-adjusted life years (DALYs) for 31 selected diseases in the European Union (EU) and European Economic Area (EEA). **Methods:** DALYs were estimated using an incidence-based and pathogen-based approach. Incidence was estimated through assessment of data availability and quality, and a correction was applied for under-estimation. Calculation of DALYs was performed with the BCoDE software toolkit without applying time discounting and age-weighting. **Results:** We estimated that one in 14 inhabitants experienced an infectious disease episode for a total burden of 1.38 million DALYs (95% uncertainty interval (UI): 1.25–1.5) between 2009 and 2013; 76% of which was related to the acute phase of the infection and its short-term complications. Influenza had the highest burden (30% of the total burden), followed by tuberculosis, human immunodeficiency virus (HIV) infection/AIDS and invasive pneumococcal disease (IPD). Men had the highest burden measured in DALYs (60% of the total), adults 65 years of age and over had 24% and children less than 5 years of age had 11%. Age group-specific burden showed that infants (less than 1 year of age) and elderly people (80 years of age and over) experienced the highest burden. **Conclusions:** These results provide baseline estimates for evaluating infectious disease prevention and control strategies. The study promotes an evidence-based approach to describing population health and assessing surveillance data availability and quality, and provides information for the planning and prioritisation of limited resources in infectious disease prevention and control.

## Introduction

Countries of the European Union (EU) and European Economic Area (EEA) increasingly face the challenge of how best to allocate limited resources for infectious disease prevention and control. Evidence to determine priorities is often limited and epidemiological data may be unavailable, of uncertain quality or difficult to communicate to decision makers. Burden of disease estimates, using composite health measures, provide clear and comprehensive information for transparent and accountable decision making and have the potential to play an important role in health policy formulation [1]. Numerous studies have addressed the challenge of estimating disease burden regionally, nationally and globally [2-8].

In high-income countries, the incidence of infectious diseases has decreased over the last century, but recent outbreaks of emerging and re-emerging diseases worldwide, such as severe acute respiratory syndrome (SARS), Middle East respiratory syndrome (MERS), measles, avian and pandemic influenza, chikungunya virus, Ebola virus disease (EVD) and Zika virus disease, have resulted in a renewed focus on infectious diseases [9-14]. In addition, the traditional boundaries between non-infectious diseases and infectious diseases have become blurred as increasing evidence of the aetiological role of the latter in triggering non-infectious conditions is available [15,16].

In 2006, the European Centre for Disease Prevention and Control (ECDC) commissioned a pilot disease burden study using seven selected infectious diseases in order to propose a methodology for a burden of disease study tailored towards infectious diseases and assess the feasibility of, and interest in, such an approach [17]. Based on this pilot, the Burden of Communicable Diseases in Europe (BCoDE) project was launched [18], funded by ECDC and implemented in collaboration with a European consortium led by the Dutch National Institute for Public Health and the Environment (RIVM) and consisting of academic and national health institutes from EU countries.

The main objective of the BCoDE project was to develop a methodology to assess the impact of infectious diseases on population health in EU/EEA countries. It also intended to promote an evidence-based approach to assess population health, foster analysis of surveillance data quality and availability, facilitate the communication of complex health information to decision makers, and provide a tool for the planning and prioritisation of infectious disease prevention, preparedness and control measures.

To achieve these objectives, a methodology was developed [19,20] that uses a composite health measure, the disability-adjusted life year (DALY) [21], to express the disease burden of an infectious disease in a single metric and is therefore suitable for comparing their relative burden.

In line with the overall objectives of the BCoDE project, the specific aim of the BCoDE 2009–2013 study described in this paper was to provide a baseline average annual estimate of the EU/EEA burden of selected infectious diseases surveyed by ECDC and measured in DALYs.

## Methods

### Outcome measure and disease models

The methodological framework of the BCoDE 2009–2013 study was based on the BCoDE project [19,20]. This methodology uses an incidence-based approach with a disease progression pathway to estimate DALYs, an outcome measure that describes the impact of years lived with disability (YLD) following the onset of a disease and of years of life lost due to premature mortality (YLL) compared with a standardised life expectancy [22]. The incidence-based approach acknowledges current and future sequelae of infections, and sets the baseline for estimating the impact of prevention and control interventions. The disease progression model (i.e. outcome tree) links possible sequelae to an initial infection and allocates that future burden to the time of infection.

To calculate DALYs, the incidence of acute, symptomatic disease is a key input variable. Besides the number of symptomatic infections, computation of DALYs requires several additional age group and sex-specific variables. These variables include the risk of developing short- and long-term complications (health outcomes), their duration, and weights reflecting their severity. These variables are described through disease models or outcome trees, which represent the progression of a disease over time by ordering relevant health outcomes following infection and illustrating their conditional dependency [19,20].

To determine the life expectancy at age of death, we used the same standard reference life table as the Global Burden of Disease Study 2010 (GBD 2010) [23]. Disability weights were selected from the set developed by the European disability weight project [24]. Outcome trees, their parameters and literature reviews for each disease included in this study are described in the BCoDE toolkit, version 1.2 [25] and are available in Supplement 1. No age-weighting and time-discounting was applied.

### Selection of communicable diseases

Diseases for inclusion in the present BCoDE 2009–2013 study were selected from those listed in Decision 2119/98/EC with amendments, which fall under the mandate of ECDC as part of its responsibilities for epidemiological surveillance in support of the identification, assessment and communication of threats to health due to communicable diseases in the EU/EEA countries [26]. The selection criteria were data availability, incidence, outbreak potential and whether the disease is preventable with widely used vaccines (Supplement 2). Final disease selections were made by an ad hoc working group of the ECDC Advisory Forum, a board of experts from EU/EEA countries advising the ECDC Director [27].

### Study population and European Union/European Economic Area countries included

Results represent the burden of infectious diseases in all of the EU/EEA countries, except for Croatia, which joined the EU in 2012. However, due to the wide variability of data availability and/or quality across countries and in order to balance data quality and representativeness, for some diseases the estimation was based on a subset of countries. Details are available in Supplement 3. Reasons for excluding countries included data availability (e.g. countries not reporting surveillance data to ECDC) and data completeness (e.g. countries reporting only aggregate or sentinel-based surveillance data but with the denominator population being unreported or unknown). Age group and sex-specific demographic data were obtained from the Eurostat database, 2011 [28].

### Estimation of annual number of cases

Cases of diseases notified to ECDC through The European Surveillance System (TESSy), a database of communicable diseases cases in EU/EEA countries, were used as the main data source for estimating incidence of acute infections. In order to remove the effect of large fluctuations in incidence data, for example that because of seasonality of disease or outbreaks, notified cases during five years, 2009 to 2013, were averaged to obtain an annual notified number of incident cases.

The annual number of cases was estimated in a step-wise approach, generally by multiplying the age group and sex-specific number of cases notified to ECDC by a multiplication factor adjusting for underestimation [29]. For full details see Supplement 3 and Table 1. In order to determine the most suitable multiplication factors, we reviewed the available TESSy data.

**Table 1:** Annual notification rate of selected infectious diseases, multiplication factors adjusting for under-estimation, and countries included in the estimation of DALYs, EU/EEA countries, 2009–2013

Infectious disease	EU/EEA annual notification of confirmed cases per 100,000 population^a^					Multiplication factors adjusting for under-estimation^b,c^	EU/EEA population included in the estimation of DALYs	
	2009	2010	2011	2012	2013		Countries represented^d^	Percent of EU/EEA population (%)
Campylobacteriosis^e^	49.64	53.53	55.43	52.62	52.30	NA	Austria, Denmark, Finland, France, Ireland, Italy, the Netherlands, Poland, Romania and Spain	35
Chlamydia infection^f^	189.06	178.90	178.25	184.79	184.45	No multiplication factor for perinatal chlamydia NA for acquired chlamydia	All countries	100
Congenital toxoplasmosis^f,g,h^	10.04	7.87	6.18	4.16	6.23	NA	All countries	100
Cryptosporidiosis	2.77	2.36	2.02	3.19	2.32	8.2 to 13.9	Belgium, Finland, Germany, Hungary, Ireland, Latvia, Spain, Sweden and UK	46
Diphtheria	NS	NS	NS	0.01	NS	2	Belgium, Finland, France, Germany, Latvia, Lithuania, the Netherlands, Sweden and UK	50
Giardiasis	5.79	6.06	5.65	5.46	5.50	14 (4 to 49)	Austria, Belgium, Cyprus, Czech Republic, Estonia, Finland, Germany, Hungary, Iceland, Ireland, Latvia, Lithuania, Luxembourg, Malta, Norway, Romania, Slovakia, Slovenia, Spain, Sweden and UK	51
Gonorrhoea	8.88	8.71	10.49	12.55	16.99	1.01 to 3.86 for acquired and congenital	Cyprus, Czech Republic, Denmark, Estonia, Finland, Iceland, Ireland, Italy, Latvia, Lithuania, Luxemburg, Malta, Norway, Portugal, Romania, Slovakia, Slovenia, Sweden (and UK for acquired cases)	42 (acquired) 41 (congenital)
Hepatitis A	3.52	2.70	2.55	2.65	2.48	4.5 (3.7 to 5.6)	All countries except Bulgaria, Lithuania, Latvia and Poland	90
Acute hepatitis B	0.80	0.80	0.70	0.70	0.70	1 to 6.6	Austria, Czech Republic, Denmark, Estonia, Finland, France, Germany, Greece, Hungary, Ireland, Italy, the Netherlands, Norway, Romania, Slovakia, Slovenia, Sweden and UK	76
Acute hepatitis C	0.30	0.70	0.50	0.60	0.50	NA	NA	NA
Human immunodeficiency virus infection/AIDS^i^	6.60	6.50	6.50	6.60	6.30	1.01 to 1.59	All countries except Italy	89
Influenza^f^	NAv	NAv	NAv	NAv	NAv	NA	All countries	100
Invasive *Haemophilus influenzae* disease	0.40	0.41	0.46	0.47	0.49	1.41 (1.35 to 1.52) for France 2.27 (2.17 to 2.44) for all other countries	All countries except Belgium and Bulgaria	89
Invasive meningococcal disease	0.91	0.75	0.81	0.73	0.71	1.0 to 1.14	All countries except Bulgaria	99
Invasive pneumococcal disease	4.39^i^	5.17	4.88	5.04	5.01	Depending on country surveillance system sensitivity: 1 to 2.5	Cyprus, Czech Republic, Denmark, Finland, Iceland, Ireland, Lithuania, Malta, Norway, Slovakia, Slovenia and Sweden	11
Legionnaires’ disease	1.10	1.16	0.88	1.06	1.06	Depending on country surveillance system sensitivity: 1 to 3.03 1 to 7.69 1 to 60.24	All countries	100
Listeriosis	0.42	0.42	0.36	0.42	0.44	1.7 (1.1 to 2.3) for acquired and perinatal	Acquired listeriosis: all countries except Bulgaria and Lithuania Perinatal listeriosis: Austria, Cyprus, France, Greece, Hungary, Italy, Latvia, the Netherlands, Poland, Romania, Slovakia, Sweden and UK	98 (acquired) 67 (congenital)
Measles	13.91	68.59	63.00	22.18	20.96	1.5 for outbreak year to 2.5 for non-outbreak year	All countries	100
Mumps	4.90	3.32	3.50	5.40	5.86	4.57 to 6.99	All countries except Belgium, France and Germany	70
Pertussis^f^	5.80	4.44	5.50	11.65	5.92	NA	All countries	100
Poliomyelitis	0.00	0.00	0.00	0.00	0.00	NA	All countries	100
Q Fever^i^	0.88	0.35	0.20	0.16	0.17	5.04	All countries except Austria, Belgium, Bulgaria, Denmark and Italy	76
Rabies^i^	NS	NS	NS	NS	NS	NA	All countries	100
Rubella	4.81	2.25	15.48	76.50	140.30	10 for acquired rubella 2 to 3.57 for congenital rubella syndrome	Acquired rubella: Austria, Bulgaria, Cyprus, Czech Republic, Estonia, Finland, Greece, Hungary, Iceland, Ireland, Italy, Latvia, Lithuania, Luxembourg, Malta, the Netherlands, Norway, Poland, Portugal, Romania, Slovakia, Slovenia, Spain, Sweden and UK Congenital rubella syndrome: all countries except Austria	68 (acquired) 98 (congenital)
Salmonellosis^e^	26.34	24.67	23.53	23.19	21.37	NA	Austria, Denmark, Finland, France, Greece, Ireland, Italy, the Netherlands, Poland, Romania, Spain, Sweden and UK	62
Shigellosis	1.88	1.82	1.76	1.53	1.37	18.3 (2.9 to 39.5)	All countries except Bulgaria, Lithuania, Luxembourg and Poland	91
Shiga toxin/verocytotoxin-producing *Escherichia coli* (STEC/VTEC) infection^i^	0.84	0.84	2.20	1.28	1.37	26.68 (1.6 to 109.7)	All countries except Bulgaria, Lithuania and Italy	86
Syphilis	4.43	4.20	4.61	4.63	4.93	1.01 to 3.86 for acquired syphilis 1 for congenital syphilis	Acquired syphilis: Czech Republic, Estonia, France, Ireland, Latvia, Lithuania, Malta, the Netherlands, Norway, Portugal, Romania, Slovakia, Slovenia and Sweden Congenital syphilis: Bulgaria, Cyprus, Czech Republic, Estonia, Germany, Greece, Hungary, Iceland, Ireland, Italy, Latvia, Lithuania, Luxembourg, Malta, Norway, Poland, Portugal, Romania, Slovakia, Slovenia, Spain, Sweden and UK	31 (acquired) 75 (congenital)
Tetanus	0.03	0.03	0.04	0.03	0.02	1.41 to 2.78	All countries except Finland and Germany	83
Tick-borne encephalitis^j^	NAv	NAv	NAv	0.54	0.71	3.33 to 5^k^	Austria, Czech Republic, Estonia, Finland, France, Germany, Greece, Hungary, Ireland, Latvia, Lithuania, Norway, Poland, Romania, Slovakia, Slovenia, Spain, Sweden and UK	78
Tuberculosis	15.87	15.00	14.32	13.50	12.66	Country-specific depending on country surveillance system sensitivity^l^	All countries	100
Variant Creutzfeldt–Jakob disease	NS	NS	NS	NS	NS	NA	All countries	100

DALYs: disability-adjusted life years; EU/EEA: European Union/European Economic Area; NA: not applicable; NAv: not available; NS: not specified (< 0.01); UK: United Kingdom.

^a^ Except where indicated, based on the European Centre for Disease Prevention and Control (ECDC) surveillance atlas of infectious diseases (accessed 1 August 2016) [59].

^b^ One number only corresponds to a point estimate, two numbers constitute a range and three numbers refer to a most likely value, lower and upper bound. See Supplement 3 for more details.

^c^ Under-estimation is the combination of under-reporting, those cases that are not reported to the surveillance system, and under-ascertainment, those cases that did not access the healthcare system. See Gibbons et al. [29].

^d^ All countries means all EU/EEA countries except for Croatia, which joined the EU in 2012.

^e^ Estimation of incidence is based on a seroincidence study [60].

^f^ Estimation of incidence is based on age group and sex-specific incidence or prevalence from published literature.

^g^ Notification rate per 100,000 < 1-year-of-age population.

^h^ Acquired form is not notifiable to ECDC.

^i^ Based on ECDC annual epidemiological reports [61].

^j^ 2012 and 2013 notified data only as disease was not previously notifiable to ECDC.

^k^ Notification of cases in meningo-encephalitic phase only; therefore, data are adjusted in order to estimate the number of symptomatic cases.

^l^ Based on the ECDC/World Health Organization Regional Office for Europe report, Tuberculosis surveillance and monitoring in Europe 2015 [62].

The first step involved determining the availability of notification data: which countries reported and for which years. Countries not reporting or reporting limited information on sex and age of cases data were excluded from the study. The second step involved reviewing annual notification rates separately for each country, and the third step involved comparing the average rates across different countries. During these steps, together with ECDC surveillance experts, we considered surveillance systems’ characteristics, including case definition, case-based vs aggregate reporting, compulsory vs voluntary reporting, comprehensive vs sentinel surveillance and whether or not the surveillance system had national coverage. Notification rates were also reviewed in relation to epidemiological circumstances (e.g. outbreaks and disease exposure), reporting practices, healthcare providers’ awareness, and healthcare system characteristics.

For a number of diseases, i.e. campylobacteriosis, chlamydia infection, congenital toxoplasmosis, influenza, pertussis and salmonellosis, it was concluded that it was not possible to estimate the incidence from notified data and alternative methods were applied (see Supplement 3). In particular, no published large community study was found for influenza except for the results of the Flu Watch cohort study in the United Kingdom [30], which we chose as the main data source to model the incidence of influenza in EU/EEA countries.

All the approaches above were explored in order to estimate the incidence of acute hepatitis C virus (HCV) infection in the general population. However, only published serological studies based on limited populations at risk were found, which would have introduced an unmeasurable bias and uncertainty and would not have allowed to estimate the incidence in the total population. Therefore, we excluded HCV infection from our disease burden estimation as no reliable data on the annual incidence of acute HCV was identified.

### Computational analysis and uncertainty

For each disease, a model was generated using the BCoDE toolkit. Within each model, the age group-specific and sex-specific annual number of cases, multiplication factors adjusting for underestimation and population were inserted in the software. Uncertainty intervals (UI) were expressed as Uniform (2 values) or Project Evaluation and Review Techniques (PERT) (3 values) distributions; we ran the models at 10,000 iterations of the Monte Carlo simulations, without time discounting and age-weighting. For each disease, results included DALYs per case and the following per 100,000 population: incidence, deaths, YLL, YLD and DALYs. For all the outputs, we showed the median and the 95% UI.

### Ethics statement

The BCoDE 2009–2013 study used a combination of aggregate health information (i.e. without personal identifiers) notified to ECDC through TESSy and information stemming from the scientific literature; therefore, informed consent was not required. Other information included in the study was drawn from published literature.

## Results

We estimated that between 2009 and 2013, the selected 31 infectious diseases accounted for 7,577 cases per 100,000 population per year (95% UI: 6,445–8,141) and there were 9.67 deaths per 100,000 population annually (95% UI: 8.47–10.3) (Table 2). Considering the EU/EEA population in 2011, these numbers would correspond to 37,784,603 cases (95% UI: 32,139,602–40,597,130) and 48,222 deaths (95% UI: 42,238–51,364).

**Table 2:** Ranking of selected infectious diseases according to annual DALYs per 100,000 population, EU/EEA countries, 2009–2013

Infectious disease	Median (95% uncertainty interval)^a^						% of total DALYs
	Incidence per 100,000 population	Deaths per 100,000 population	DALYs per case	YLD per 100,000 population	YLL per 100,000 population	DALY per 100,000 population	
Influenza	5,887 (5,544–6,223)	5.89 (5.54–6.22)	0.01	5.42 (4.73–6.16)	76.3 (71.9–80.7)	81.8 (76.9–86.5)	29.8
Tuberculosis	14.9 (14.7–15.2)	1.10 (1.08–1.12)	3.58 (3.55–3.62)	9.20 (8.98–9.43)	44.3 (43.5–45.1)	53.5 (52.5–54.4)	19.5
Human immunodeficiency virus infection	7.99 (7.44–8.55)	0.15 (0.13–0.16)	6.03 (5.86–6.20)	43.1 (39.7–46.4)	5.13 (4.53–5.64)	48.2 (44.5–51.9)	17.5
Invasive pneumococcal disease	11.0 (10.7–11.2)	1.18 (1.15–1.21)	2.74 (2.71–2.77)	2.49 (2.25–2.73)	27.6 (26.9–28.2)	30.1 (29.3–30.8)	10.9
Legionnaires’ disease	3.40 (2.77–4.01)	0.37 (0.30–0.45)	3.04 (2.73–3.36)	0.02 (0.02–0.03)	10.3 (8.21–12.4)	10.3 (8.23–12.4)	3.75
Campylobacteriosis	654 (599–707)	0.18 (0.13–0.23)	0.01	3.25 (2.73–3.87)	5.03 (3.59–6.58)	8.28 (6.68–10.0)	3.01
Hepatitis B	2.84 (2.29–3.40)	0.15 (0.09–0.21)	2.79 (1.46–4.45)	0.49 (0.30–0.72)	7.37 (3.85–11.7)	7.86 (4.19–12.2)	2.86
Invasive *Haemophilus influenzae* disease	1.52 (1.51–1.53)	0.17	3.43 (3.39–3.47)	0.28 (0.24–0.31)	4.94 (4.88–5.00)	5.22 (5.15–5.29)	1.90
Invasive meningococcal disease	0.85 (0.83–0.86)	0.07	5.64 (5.59–5.70)	0.39 (0.35–0.44)	4.39 (4.31–4.48)	4.78 (4.68–4.88)	1.74
Chlamydia infection	186 (124–259)	< 0.01	0.02 (0.01–0.05)	4.62 (2.16–9.0)	Negligible	4.63 (2.16–9.03)	1.68
Salmonellosis	211 (208–214)	0.16 (0.15–0.17)	0.02	0.86 (0.74–1.01)	3.11 (2.85–3.36)	3.97 (3.68–4.25)	1.44
Pertussis	263 (211–317)	0.02	0.01	2.04 (1.59–2.56)	1.28 (1.14–1.45)	3.33 (2.78–3.94)	1.21
Shiga toxin/verocytotoxin-producing Escherichia coli (STEC/VTEC) infection	48.1 (36.2–59.4)	0.05 (0.04–0.07)	0.05 (0.05–0.06)	0.62 (0.49–0.76)	1.98 (1.56–2.44)	2.59 (2.05–3.21)	0.94
Listeriosis	0.56 (0.52–0.59)	0.08 (0.08–0.09)	3.65 (3.52–3.79)	0.20 (0.15–0.25)	1.84 (1.74–1.94)	2.04 (1.92–2.16)	0.74
Rubella	51.6	< 0.01	0.02 (0.01–0.02)	0.55 (0.39–0.74)	0.37 (0.29–0.45)	0.92 (0.71–1.15)	0.33
Gonorrhoea	34.2 (24.4–44.2)	< 0.01	0.02 (0.01–0.04)	0.77 (0.49–1.24)	0.01 (0.01–0.02)	0.78 (0.50–1.26)	0.28
Hepatitis A	10.0 (9.67–10.4)	0.02	0.07 (0.06–0.08)	0.14 (0.11–0.17)	0.58 (0.51–0.66)	0.72 (0.64–0.80)	0.26
Tick-borne encephalitis	3.00 (2.87–3.13)	< 0.01	0.23 (0.22–0.24)	0.46 (0.43–0.49)	0.23 (0.22–0.25)	0.69 (0.65–0.74)	0.25
Shigellosis	27.0 (23.4–30.7)	0.01 (0.01–0.02)	0.03 (0.02–0.03)	0.09 (0.08–0.11)	0.59 (0.41–0.82)	0.68 (0.49–0.93)	0.25
Measles	7.46	< 0.01	0.08 (0.07–0.08)	0.14 (0.11–0.17)	0.42 (0.38–0.46)	0.56 (0.51–0.61)	0.20
Congenital toxoplasmosis	0.19 (0.11–0.28)	< 0.01	2.42 (1.92–3.05)	0.34 (0.17–0.56)	0.12 (0.06–0.19)	0.46 (0.24–0.73)	0.17
Giardiasis	88.9 (75.0–104)	< 0.01	< 0.01	0.36 (0.30–0.43)	0.05 (0.04–0.06)	0.41 (0.34–0.48)	0.15
Q fever	1.58	< 0.01	0.20 (0.16–0.23)	< 0.01	0.31 (0.25–0.36)	0.31 (0.26–0.37)	0.11
Tetanus	0.06 (0.05–0.07)	< 0.01	2.02 (1.91–2.15)	< 0.01	0.12 (0.11–0.13)	0.12 (0.11–0.13)	0.04
Mumps	24.2 (22.6–25.8)	< 0.01	< 0.01	0.07 (0.06–0.07)	0.02	0.09 (0.08–0.10)	0.03
Cryptosporidiosis	34.7 (32.3–37.1)	< 0.01	< 0.01	0.03 (0.02–0.03)	0.06 (0.05–0.06)	0.08 (0.08–0.09)	0.03
Syphilis	2.04 (1.68–2.38)	< 0.01	0.04 (0.04–0.05)	0.04 (0.03–0.04)	0.05	0.08 (0.08–0.09)	0.03
variant Creutzfeldt–Jakob disease	< 0.01	< 0.01	48.6 (48.4–48.8)	< 0.01	0.04	0.04	0.01
Diphtheria	0.02	< 0.01	1.16	< 0.01	0.02	0.02	0.01
Rabies	< 0.01	< 0.01	52.1	< 0.01	0.01	0.01	< 0.01
**Total**	**7,577 (6,445–8,141)**	**9.67 (8.47–10.3)**	**NA**	**75.9 (66.0–87.0)**	**196 (181–213)**	**273 (249–299)**	**100**

DALYs: disability-adjusted life years; EU/EEA: European Union/European Economic Area; YLD: years lived with disability: YLL: years of life lost; NA: not applicable.

^a^ Median and 95% uncertainty interval as estimated from the Burden of Communicable Diseases in Europe (BCoDE) toolkit (10,000 Monte Carlo simulations).

Values < 0.01 are not specified. Uncertainties that deviate less than 0.01 from the median are not specified.

**Figure 1:**
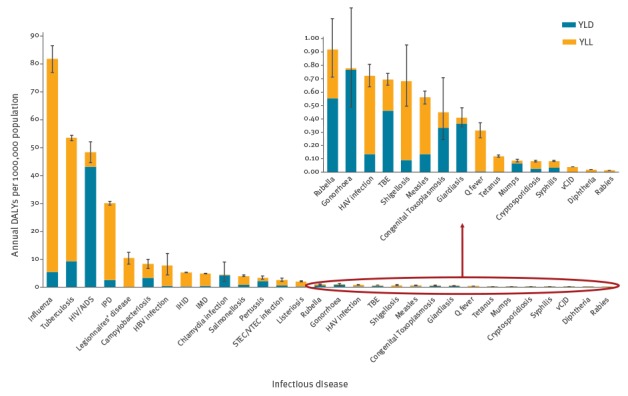
Median annual DALYs per 100,000 population for selected infectious diseases, EU/EEA countries, 2009–2013

The annual burden of the infectious diseases included in our study was 275 DALYs per 100,000 population (95% UI: 249–299). The disease with the highest burden was influenza, with 81.8 DALYs per 100,000 population (95% UI: 76.9–86.5), followed by tuberculosis (TB), human immunodeficiency virus (HIV) infection/AIDS and invasive pneumococcal disease (IPD) with 53.5 (95% UI: 52.5–54.4), 48.2 (95% UI: 44.5–51.9) and 30.1 (95% UI: 29.3–30.8 DALYs per 100,000 population respectively (Table 2, Figure 1). These four top-ranking infections accounted for 78% of the total burden of communicable diseases in EU/EEA countries.

EU/EEA: European Union/European Economic Area; HAV: Hepatitis A virus; HBV: Hepatitis B virus; HIV/AIDS: Human immunodeficiency virus infection; IHID: Invasive *Haemophilus influenzae* disease; IMD: Invasive meningococcal disease; IPD: Invasive pneumococcal disease; STEC/VTEC: Shiga toxin/verocytotoxin-producing *Escherichia coli*; TBE: Tick-borne encephalitis; vCJD: variant Creutzfeldt–Jakob disease; YLD: years lived with disability; YLL: years of life lost due to premature mortality.

The error bars indicate the 95% uncertainty intervals.

**Figure 2:**
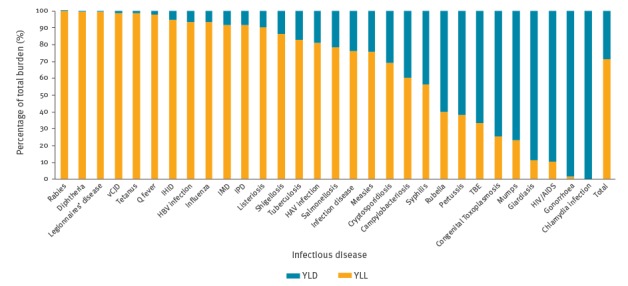
Relative contribution of YLL and YLD to the total burden of selected infectious diseases, EU/EEA countries, 2009–2013

Legionnaires’ disease, campylobacteriosis and hepatitis B had a significantly lower burden compared to the four diseases discussed above. Invasive *Haemophilus influenzae* disease, invasive meningococcal disease, chlamydia, salmonellosis, pertussis and Shiga toxin/verocytotoxin-producing *Escherichia coli* (STEC/VTEC) infection had an even lower burden. The remaining diseases were ranked with a significantly lower burden. YLL accounted for 71% of the total burden (Figure 2).

EU/EEA: European Union/European Economic Area; HAV: Hepatitis A virus; HBV: Hepatitis B virus; HIV/AIDS: Human immunodeficiency virus infection; IHID: Invasive *Haemophilus influenzae* disease; IMD: Invasive meningococcal disease; IPD: Invasive pneumococcal disease; STEC/VTEC: Shiga toxin/verocytotoxin-producing *Escherichia coli*; TBE: Tick-borne encephalitis; vCJD: variant Creutzfeldt–Jakob disease; YLD: years lived with disability; YLL: years of life lost due to premature mortality.

**Figure 3:**
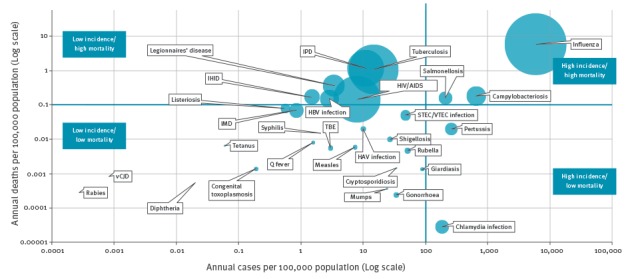
Bubble chart of the burden of selected infectious diseases in terms of mortality and incidence, EU/EEA countries, 2009–2013

Diseases with higher incidence and mortality as compared with other diseases were found to be influenza, campylobacteriosis and salmonellosis (Figure 3), although only the former has a high burden in DALYs. Pertussis and chlamydia have high incidence and low mortality, whereas TB, HIV/AIDS, IPD, Legionnaires’ disease, hepatitis B virus (HBV) infection and invasive *Haemophilus influenzae* disease (IHID) had low incidence and high mortality.

EU/EEA: European Union/European Economic Area; HAV: Hepatitis A virus; HBV: Hepatitis B virus; HIV/AIDS: Human immunodeficiency virus infection; IHID: Invasive *Haemophilus influenzae* disease; IMD: Invasive meningococcal disease; IPD: Invasive pneumococcal disease; STEC/VTEC: Shiga toxin/verocytotoxin-producing *Escherichia coli*; TBE: Tick-borne encephalitis; vCJD: variant Creutzfeldt–Jakob disease

The diameter of the bubble reflects the number of DALYs per 100,000 population per year.

### Burden of congenital infections in newborns

In terms of burden of congenital infections in the newborns, almost all the burden (97%) was attributable to toxoplasmosis, listeriosis and rubella infections (Table 3).

**Table 3:** Ranking of congenital diseases by DALYs and proportion among total congenital diseases per 100,000 newborn population, EU/EEA countries, 2009–2013

Disease	Median DALYs per 100,000 newborn population (95% uncertainty interval)^a^	DALYs due to congenital infections per 100,000 newborn population (%)
Congenital toxoplasmosis	43.6 (22.7–68.6)	35.1
Congenital rubella	42.5 (30.6–56.7)	34.6
Perinatal listeriosis	34.4 (26.2–43.4)	28.0
Congenital syphilis	2.73 (2.64–2.81)	2.22
Congenital chlamydia infection	0.09 (0.08–0.10)	0.08
Congenital gonorrhoea	< 0.01	< 0.01
**Total**	**123 (82.2–172)**	**100**

DALYs: disability-adjusted life years; EU/EEA: European Union/European Economic Area; YLD: years lived with disability: YLL: years of life lost.

^a^ Median and 95% uncertainty interval as estimated from the Burden of Communicable Diseases in Europe (BCoDE) toolkit (10,000 Monte Carlo simulations).

Values < 0.01 are not specified.

### Comparison of DALYs at the individual and population level

**Figure 4:**
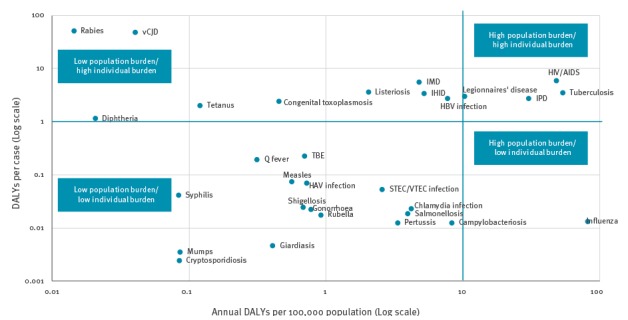
Scatterplot of the burden of selected infectious diseases in DALYs per case and DALYs per 100,000 population per year, EU/EEA countries, 2009–2013

The diseases with the highest number of DALYs per case, which represents the individual burden and to a certain extent the severity of the disease, were rabies and variant Creutzfeldt–Jakob disease, which are ultimately fatal conditions. HIV/AIDS, invasive meningococcal disease, listeriosis, TB, IHID, Legionnaires’ disease, HBV infection, IPD, congenital toxoplasmosis, tetanus and diphtheria followed, with DALYs per case ranging from 6.03 to 1.16. Diseases determined to have a high individual and population burden were Legionnaires’ disease, IPD, HIV/AIDS and TB, while influenza was determined to have a low individual but high population burden (Figure 4).

EU/EEA: European Union/European Economic Area; HAV: Hepatitis A virus; HBV: Hepatitis B virus; HIV/AIDS: Human immunodeficiency virus infection; IHID: Invasive *Haemophilus influenzae* disease; IMD: Invasive meningococcal disease; IPD: Invasive pneumococcal disease; STEC/VTEC: Shiga toxin/verocytotoxin-producing *Escherichia coli*; TBE: Tick-borne encephalitis; vCJD: variant Creutzfeldt–Jakob disease

Diseases were arbitrarily subdivided according to burden in DALYs per 100,000 population and DALYs per case.

### DALYs by sex and age

Most DALYs, around 60%, were due to infections occurring in males. Considering more detailed results presented in Supplement 4, diseases such as TB, HIV/AIDS, Legionnaires’ disease, were found to impact mostly men while chlamydia and gonorrhoea had a higher burden in women.

**Figure 5:**
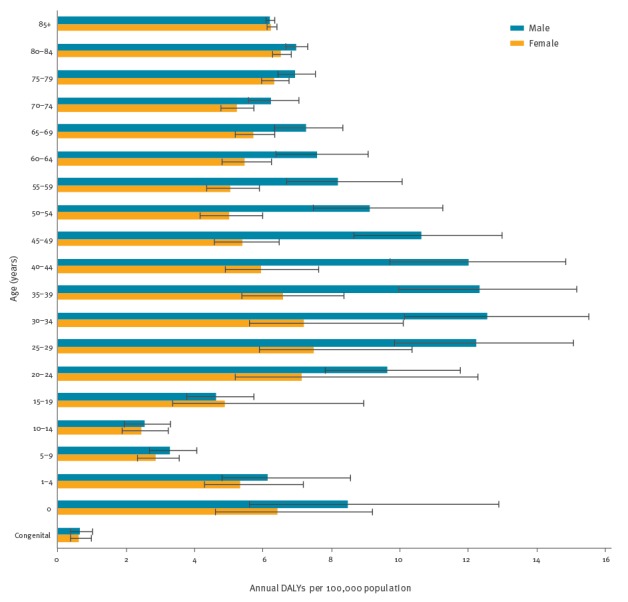
Annual total burden of selected infectious diseases by age group and sex, EU/EEA countries, 2009–2013

**Figure 6:**
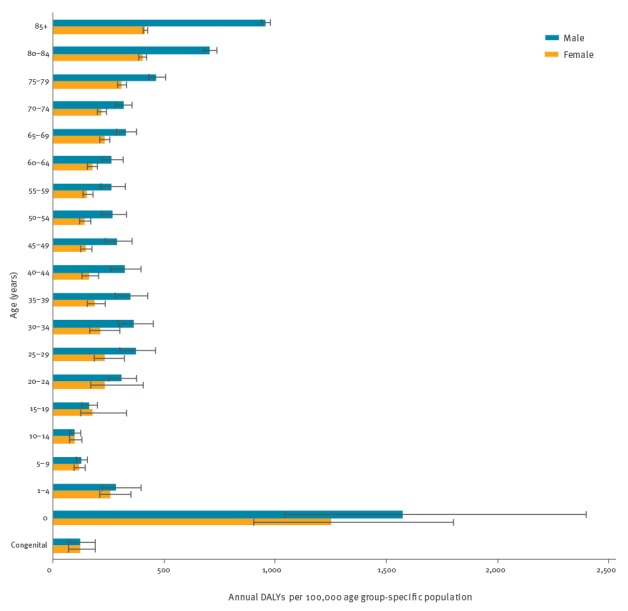
Annual age group-standardised burden of selected infectious diseases by age group and sex, EU/EEA countries, 2009–2013

When considering DALYs over the total population, 11% occurred in children less than 5 years of age, 15% in individuals less than 15 years of age and 24% in individuals aged 65 years and over (see Supplement 4); most DALYs were found in age groups between 25 and 49 year of age (Figure 5). However, when considering the age group-specific DALYs per 100,000 population of the age group, those with the highest overall burden were infants under one year of age and individuals 80 years of age and over (Figure 6).

EU/EEA: European Union/European Economic Area.

The error bars indicate the 95% uncertainty intervals.

EU/EEA: European Union/European Economic Area.

The error bars indicate the 95% uncertainty intervals.

**Figure 7:**
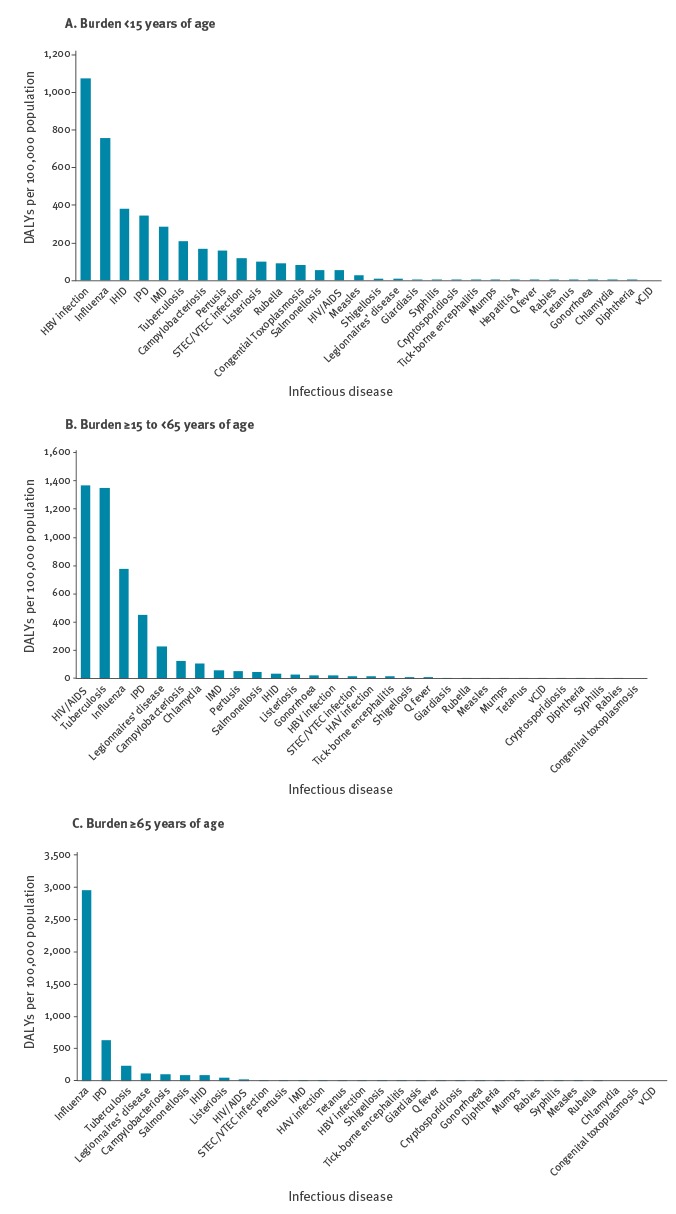
Annual age group-specific burden of selected infectious diseases by age groups < 15 years of age, 15–64 years of age and ≥ 65 years of age, EU/EEA countries, 2009–2013

Compared with the age groups of between 15 and 64 years of age (adults) and 65 years of age and over (elderly population), the total burden of disease in the population under 15 years of age is lower (Figure 7). The diseases with the highest burden in the under 15 years age group are HBV infection, influenza, IHID, IPD and invasive meningococcal disease (IMD). HIV/AIDS, TB and influenza are the diseases with the highest burden in the adult population, whereas influenza, IPD and TB have the highest impact in the elderly population.

EU/EEA: European Union/European Economic Area; HAV: Hepatitis A virus; HBV: Hepatitis B virus; HIV/AIDS: Human immunodeficiency virus infection; IHID: Invasive *Haemophilus influenzae* disease; IMD: Invasive meningococcal disease; IPD: Invasive pneumococcal disease; STEC/VTEC: Shiga toxin/verocytotoxin-producing *Escherichia coli*; TBE: Tick-borne encephalitis; vCJD: variant Creutzfeldt–Jakob disease.

### Contribution of the acute phase of the disease and of years of life lost due to premature mortality to disability-adjusted life years (DALYs)

The acute phase of diseases had the highest impact on the total burden (76%) (see Supplement 4). This was the result of the outcome trees that modelled case fatality proportions (CFP) as a direct risk to the acute infection. The high share of YLLs (72% of total DALYs, see Table 2) compared with YLDs was due to the limited amount of time lived with a disability, which is typical for infectious diseases.

### Comparison of rankings

**Figure 8:**
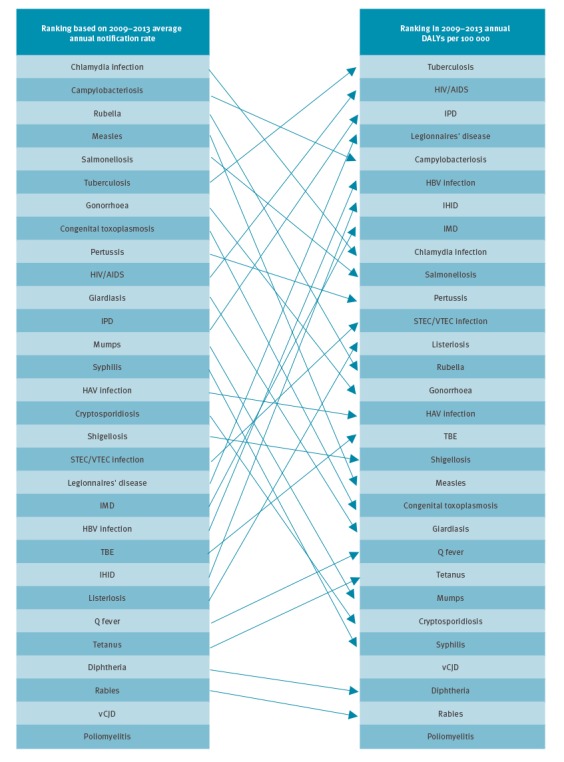
Comparison of ranking according to ECDC TESSy average annual notification rate and ranking according to estimated DALYs per 100,000 population, EU/EEA countries, 2009–2013

DALYs: disability-adjusted life years; ECDC: European Centre for Disease Prevention and Control; EU/EEA: European Union/European Economic Area; HAV: Hepatitis A virus; HBV: Hepatitis B virus; HIV/AIDS: Human immunodeficiency virus infection; IMD: Invasive meningococcal disease; IHID: Invasive *Haemophilus influenzae* disease; IPD: Invasive pneumococcal disease; STEC/VTEC: Verocytotoxigenic *Escherichia coli*; TBE: Tick-borne encephalitis; TESSy: The European Surveillance System; vCJD: variant Creutzfeldt–Jakob disease.

The final ranking of the burden of disease gives a new picture of the impact of infectious diseases when compared with notification data (Figure 8).

Influenza is not included as its notification to ECDC is not case-based.

## Discussion

This study presents the estimation of the burden of 31 selected infectious diseases in the EU/EEA in DALYs, adopting an incidence- and pathogen-based methodology and a consistent approach to surveillance and outcome data assessment. The results allow ranking of infectious diseases taking morbidity, disability and premature mortality resulting from acute infections and their sequelae into account.

The incidence-based approach chosen for this study allows for the effect of future long-term complications of a disease to be included in the calculation of DALYs, resulting in a more comprehensive estimate of the effect of prevention and control interventions [31]. Compared to a prevalence-based approach, in the incidence-based DALYs, the potential future burden avoided, for example, by vaccination as a possible intervention measure, is included [19,20,32].

We did not apply time discounting, which is generally applied in economic studies, because we did not consider there to be reasons justifying the decline of healthy life years over time. Similarly, age-weighting was also not applied because it was considered that a healthy life year should be valued equally, irrespective of the age at which it is lived or lost. Both choices are consistent with current methodologies used by the World Health Organization’s (WHO) Foodborne Disease Burden Epidemiology Reference Group (FERG) and the Global Burden of Disease studies [23,33].

Access to healthcare varies across countries but is largely universal. Although healthcare and surveillance systems vary, incidence data included in this study is mainly based on cases of disease notified to national surveillance systems and reported to TESSy during years when the reporting procedures were considered to be consolidated. Surveillance in the EU/EEA differs in terms of purpose and systems for collecting data. This study enabled a thorough review of surveillance data availability and quality for each disease and each country. As a result, this study increases our knowledge and indicates areas for improving European infectious disease surveillance.

The averaging of annual number of cases over 5 years removed the effects of large fluctuations in incidence, i.e. flattened the effects of outbreaks. However, it could still be valuable to show the effect of an outbreak given that such can cause rankings to substantially change from the baseline. For example, the burden of disease was 35.5 DALYs per 100,000 Bulgarian population per year considering the 2010 measles outbreak of just under 22,000 cases [34]. This burden would have led to this outbreak ranking fourth in our results, between HIV/AIDS and IPD.

Our study ranked influenza as the infectious disease with the highest impact on population health in the EU/EEA. Although the CFP chosen for the influenza disease model was low, the incidence was significantly higher than that of any other disease included in our study (Table 2). The main driver of the high burden of influenza is the contribution of premature mortality associated with the infection (YLL). Our study estimated a mortality of 5.89 per 100,000 population, slightly lower than the ECDC-estimated annual average influenza deaths in EU/EAA countries of 7.60 per 100,000 population (range: 1.07–15.5) within the same period based on the published figures of 38,500 deaths (range: 5,400 to 78,200) [35]. Similar mortality rates were published in national studies in the Netherlands: 3.69 to 18.8 per 100,000 population [36], 2.62 per 100,000 population [37] and 3.45 per 100,000 population [38]. Our estimated influenza mortality rates, based on the BCoDE outcome-tree method, are reasonably consistent with other published rates.

However, it is important to note the limitations of our estimation of DALYs for influenza, namely the single incidence data source, the Flu Watch cohort study in the United Kingdom, representing a limited geographical region [30] which may have a different epidemiological profile and vaccination coverage from other EU/EEA countries. However, the Southern Hemisphere Influenza and Vaccine Effectiveness Research and Surveillance project (SHIVERS) in New Zealand found a very similar incidence of symptomatic influenza (personal communication, Sue Huang and Don Bandaranayake, July 2016). Moreover, the Netherlands national burden of disease study [5] estimated the incidence of influenza from the general practitioner sentinel system [37]. Using the 8,670 DALYs/year of the study and the Eurostat population in the Netherlands in 2009, we calculate an average annual burden of 52.6 DALYs per 100,000 population in the period 2007 to 2011, placing it in line with our findings.

Our results for influenza support the recommendations of the Council of the European Union [39], reiterated by the 2015 ECDC influenza vaccination report [40], to develop a national seasonal influenza vaccination action plan and to achieve target vaccination coverage for older population groups, people with chronic medical conditions, pregnant women and healthcare workers.

Our estimation of TB mortality rate was in line with national notified deaths of TB. For example, the Eurostat mortality for TB was 1.07 per 100,000 population in the 28 EU countries in 2011 [41], very much in line with our estimated rate of 1.10 per 100,000 population. Our findings reinforce the need for increasing efforts in EU/EEA countries to eliminate TB.

HIV/AIDS has a high burden of disease in Europe despite the low mortality risk compared with the pre-antiretroviral treatment era. This is reflected in the overwhelming contribution of YLD to the total DALYs (ca 90%). As significant HIV transmission continues in Europe [42] and the high associated burden found in our study highlights the need to strengthen prevention and testing efforts. This study estimated that 0.15 deaths per 100,000 population were due to HIV/AIDS. Given our incidence-based approach, one must consider that this estimation is a projection of future mortality rates for people being infected in the time-period analysed, i.e. 2009 to 2013. The Eurostat (EU 28 countries) notified standardised death rate from HIV/AIDS went from 1.2 per 100,000 population in 2002 to 0.74 per 100,000 population in 2013 [41]. In our model, we projected a lower fatality assuming further decrease in the future due to improved treatment options, increased testing/early ascertainment of cases and increased treatment compliance.

Published data on observed number of deaths of IPD are comparable to those in our study: in our study CFP was 11% (see Supplement 4) while in a European study in 17 countries, death was reported in 9.0% to 10.6% of cases and changed according to age [43], in line with the models used for our study. Similarly, published IPD incidence and mortality estimates in the Netherlands based on sentinel surveillance and statistical estimation methods [44] reported incidence of 13.8 per 100,000 population and deaths of 1.6 per 100,000 population, which are very similar to those presented in our paper. Based on our study, most of the burden of IPD is experienced by adults over 55 years of age, although children aged under 5 years also significantly contribute to the total DALYs (see Supplement 4). These findings are relevant to discussions about vaccination strategies since, according to ECDC’s report on invasive bacterial diseases in 2012, ‘the majority of infections were caused by serotypes covered by the 13-valent pneumococcal conjugate vaccine PCV13’ [45].

Ranking of diseases can be also tailored to specific age groups as illustrated in Figure 7. It is interesting to note that all five top ranking diseases among the less than 15 years of age group are preventable through vaccination. Within the adult population, further research on the main risk groups affected by HIV/AIDS and TB, which by far are the two infections with the highest impact, would be advantageous in order to better inform intervention strategies. The elderly population is mostly affected by respiratory diseases (influenza, IPD, TB and Legionnaires’ disease) and gastro-intestinal diseases (campylobacteriosis and salmonellosis). Age-specific vaccination campaigns could help prevent the burden of influenza and IPD in particular.

Results from this study must be placed in a broader perspective. Recently, the burden of a six selected healthcare-associated infections (HAIs) was estimated in DALYs based on the BCoDE methodology [46]. Their cumulative burden of 501 DALYs per 100,000 population in that study was almost twice the one found in this one. These results imply that, among those surveyed by ECDC, HAIs represent the infections with the highest burden on European population. However, the methodological differences relating to the syndromic approach chosen for the burden of HAIs vs the pathogen-based approach of this study may limit comparing the results of the two studies. In particular, a number of diseases included in the current study may have been healthcare-associated (e.g. Legionnaires’ disease, diseases attributable to *Streptococcus pneumoniae*), leading to some degree of double-counting [47,48]. However, this would likely be limited given that other diseases included in the present study may be uncommon causes of HAIs (e.g. infections due to *Neisseria meningitidis*, TB, hepatitis A and B and invasive meningococcal disease) [47,48].

The 2013 Global Burden of Disease Study (GBD 2013) estimated DALYs for a large number of diseases [7]. By downloading GBD 2013 country-specific estimates from the Global Health Data Exchange (GHDx) website and totalling the DALYs for 2013, we were able to estimate the EU/EEA burden of several infectious diseases included in our project. HIV/AIDS and TB overwhelmingly ranked higher than other infectious diseases in both studies. However, the GBD 2013 calculated prevalent DALYs, assuming a steady state and not taking into account the projected future burden, so comparisons must be made with caution.

The Ontario Burden of Infectious Disease Study (ONBOIDS) [4] used a comparable incidence-based DALY estimation approach as applied in our study. Similarly to our findings, the ONBOIDS found that infections caused by *Streptococcus pneumoniae*, HIV and influenza virus had high burden. Differences in ranking of diseases might be explained by differences in, for example, case definitions and disability weights. Epidemiological differences should also be considered, as for example, the incidence of TB is higher in EU/EEA countries than in Ontario.

Another burden of disease study based on incidence is the World Health Organization (WHO) Food-borne Disease Burden Epidemiology Reference Group (FERG) [8,49]. Considering the overlapping diseases, the diseases with the highest burden based on the published results for WHO European Region EUR A of that study and the results of this study were campylobacteriosis, salmonellosis and listeriosis. The only difference, the burden of STEC/VTEC, might be due to the higher risk of developing haemolytic uraemic syndrome (HUS) and end-stage renal disease (ESRD) in the BCoDE toolkit disease progression model.

### Strength and limitations

One strength of our study is that it is based on a rigorous assessment of national surveillance systems, which provided important information on sensitivity; where possible, notified data for a disease was adjusted specifically for each country. For example, country multiplication factors based on a self-reported survey of the national sensitivity towards IPD surveillance [50] were applied to the notification data and DALYs were estimated using the resulting cumulative number of cases. In other instances, such as for Legionnaires’ disease, countries were grouped according to ECDC disease programme expert opinion into higher, intermediate and lower surveillance system sensitivity and different multiplication factors were then applied for each sensitivity group. The sensitivity of surveillance systems does not only depend on their intrinsic characteristics; systems are also prone to temporary changes, for example, during outbreaks, when increased awareness might also increase willingness and capacity to detect and report cases. For the estimation of the incidence of measles, for example, notification of cases from countries and years experiencing an outbreak were adjusted with a multiplication factor of 1.5 [51], as opposed to 2.5 for other countries and years [52,53].

Another strength of this study is that it captures the different risks of developing sequelae or death according to age group. Examples include the age group-specific risk of developing HUS after STEC/VTEC infection, a crucial step towards the estimation of its burden, and the redistribution of the CFP of salmonellosis, campylobacteriosis and influenza according to observed age-specific mortality data in order not to overestimate the number of deaths in younger age groups [25].

Disability weights included in the disease models are derived from a study performed in Europe and thus have the potential to better reflect the preferences and values of the EU/EEA population [24]. Most infectious diseases cause temporary mild disabilities; it is important to note that according to the methodology used to estimate disability weights, these may differ substantially for similar health states [54].

A further strength is that the freely accessible and transparent methodology, parameters and variables of this study allow for reproducing the estimates and making comparisons with results from similar studies. For example, the burden of infectious diseases in the Netherlands is a national study that was based on the BCoDE project methodology with national adaptations [5].

A number of limitations need to be taken into account when interpreting the results of this study. First, the selection of diseases was limited to those included in Decision 2119/98/EC with amendments. This list does not include other infectious diseases with a potentially significant burden in EU/EEA countries, such as infections with human papillomavirus (HPV), *Helicobacter pylori*, rotavirus, norovirus and human respiratory syncytial virus (HRSV).

Second, multiplication factors adjusting for underestimation of notified data were selected from information found in the literature. Few country-specific multiplication factors were available and ranges based on the limited number of published studies were applied consistently across EU/EEA countries. Moreover, multiplication factors were not adjusted for different age groups, although some diseases causing diarrhoea, such as salmonellosis for instance, had high notification rates in children. This may be due to a testing bias, i.e. children may be tested more often, or to their reduced immunity or to higher exposure. Regardless of the reason, This means that there is a risk that the results may be underestimated or overestimated.

Third, the disease models (outcome trees) in the BCoDE toolkit are based on several assumptions [55]. Variables for each model parameter represent the available information in the literature and the age-specific risk of developing a certain sequelae or death was often not available. Outcome trees were developed considering the incidence of disease and the risks of developing sequelae as currently observed in EU/EEA countries. Therefore, treatment and preventive measures were implicitly considered and this should be taken into account when interpreting the results. For example, vaccine-preventable diseases (VPDs) with high coverage had a lower burden of disease, but they had the potential to substantially increase their burden and their resulting position in the final ranking during outbreaks. In addition, the disease models included in this study are static and do not consider future infection transmissions. Dynamic models, such as SIR compartmental models for infection transmission, should be developed when assessing the impact of prevention and control interventions.

Fourth, the probability of developing sequelae or death were estimated based on the limited information in the literature, except in some cases where information was derived from surveillance data (Supplement 1), and considered the competing risks of dying or developing complications to the extent possible. At older ages, for example, co-morbidities may worsen the severity of a given infectious disease, suggesting modification of disability weights or the need to consider the attributable fraction due to the infections as opposed to the other underlying condition.

Fifth, the burden of HBV was based on the average annual number of acute infections but like other long-term disease progression pathways, other subsequent stages of the disease have an impact on population health. It would be beneficial to complement the incidence-based HBV results with prevalence studies given that our burden of HBV estimates do not consider prevalent long-term complicated cases.

The methodology and results in this study are based on a fully transparent and reproducible approach. We believe that the burden of disease methodology described in this study provides a clear and comprehensive view on the impact of infectious diseases on population health.

## Conclusion

Calculation of DALYs through incidence-based disease progression models represents a comprehensive approach suitable for infectious diseases and provides useful information for prioritisation and planning in public health, among other purposes. For example, a recent Scientific Opinion by the European Food Safety Authority recommended using the BCoDE approach for ranking risks [56]. Another example is the Slovenian national estimation of the burden of tick-borne encephalitis that identified age groups with the highest DALYs in order to inform vaccination strategies [57].

However, as quantitative results alone might not fully encompass all unknowns, uncertainties and variability [58] other dimensions of health should be considered. Burden of disease measured in DALYs could be integrated with risk-ranking methodologies such as multi-criteria decision analysis (MCDA).

That being said, this study provides useful information for planning and prioritising surveillance strategies and intervention options aimed at preventing and controlling infectious diseases as the estimates provide a useful picture of the impact of infectious diseases in EU/EEA countries. The findings will help to inform assessment of the impact of epidemics and of public health interventions.

## References

[1] Lopez AD, Mathers CD, Ezzati M, Jamison DT, Murray CJL, editors. Global Burden of Disease and Risk Factors. New York: Oxford University Press; 2006.

[2] Stevens G Dias RH Thomas KJ Rivera JA Carvalho N Barquera S Characterizing the epidemiological transition in Mexico: national and subnational burden of diseases, injuries, and risk factors. PLoS Med. 2008;5(6):e125. 10.1371/journal.pmed.0050125 18563960 PMC2429945

[3] Public Health Group of the Department of Human Services. Victorian Burden of Disease Study: Mortality and morbidity in 2001. Melbourne: Victoria Government Department of Human Services; 2005. Available from: https://www2.health.vic.gov.au/public-health/population-health-systems/health-status-of-victorians/composite-data-and-reports-on-the-health-of-victorians/burden-of-disease

[4] Kwong JC Ratnasingham S Campitelli MA Daneman N Deeks SL Manuel DG The impact of infection on population health: results of the Ontario burden of infectious diseases study. PLoS One. 2012;7(9):e44103. 10.1371/journal.pone.0044103 22962601 PMC3433488

[5] van Lier A McDonald SA Bouwknegt M Kretzschmar ME Havelaar AH Mangen MJ Disease Burden of 32 Infectious Diseases in the Netherlands, 2007-2011. PLoS One. 2016;11(4):e0153106. 10.1371/journal.pone.0153106 27097024 PMC4838234

[6] Murray CJ Vos T Lozano R Naghavi M Flaxman AD Michaud C Disability-adjusted life years (DALYs) for 291 diseases and injuries in 21 regions, 1990-2010: a systematic analysis for the Global Burden of Disease Study 2010. Lancet. 2012;380(9859):2197-223. 10.1016/S0140-6736(12)61689-4 23245608

[7] Vos T Barber RM Bell B Bertozzi-Villa A Biryukov S Bolliger I Global, regional, and national incidence, prevalence, and years lived with disability for 301 acute and chronic diseases and injuries in 188 countries, 1990-2013: a systematic analysis for the Global Burden of Disease Study 2013. Lancet. 2015;386(9995):743-800. 10.1016/S0140-6736(15)60692-4 26063472 PMC4561509

[8] Havelaar AH Kirk MD Torgerson PR Gibb HJ Hald T Lake RJ World Health Organization Global Estimates and Regional Comparisons of the Burden of Foodborne Disease in 2010. PLoS Med. 2015;12(12):e1001923. 10.1371/journal.pmed.1001923 26633896 PMC4668832

[9] Morens DM Folkers GK Fauci AS The challenge of emerging and re-emerging infectious diseases. Nature. 2004;430(6996):242-9. 10.1038/nature02759 15241422 PMC7094993

[10] Jones KE Patel NG Levy MA Storeygard A Balk D Gittleman JL Global trends in emerging infectious diseases. Nature. 2008;451(7181):990-3. 10.1038/nature06536 18288193 PMC5960580

[11] Quaglio G Demotes-Mainard J Loddenkemper R Emerging and re-emerging infectious diseases: a continuous challenge for Europe. Eur Respir J. 2012;40(6):1312-4. 10.1183/09031936.00111712 23204016

[12] Kaasik-Aaslav K Coulombier D The tail of the epidemic and the challenge of tracing the very last Ebola case. Euro Surveill. 2015;20(12):21075. 10.2807/1560-7917.ES2015.20.12.21075 25846487

[13] European Centre for Disease Prevention and Control (ECDC). Rapid Risk Assessment. Zika virus disease epidemic. Seventh update, 8 July 2016. Stockholm: ECDC; 2016. Available from: https://ecdc.europa.eu/sites/portal/files/media/en/publications/Publications/RRA-Zika-virus%20epidemic-seventh-update-final.pdf

[14] Rezza G Nicoletti L Angelini R Romi R Finarelli AC Panning M Infection with chikungunya virus in Italy: an outbreak in a temperate region. Lancet. 2007;370(9602):1840-6. 10.1016/S0140-6736(07)61779-6 18061059

[15] de Martel C Ferlay J Franceschi S Vignat J Bray F Forman D Global burden of cancers attributable to infections in 2008: a review and synthetic analysis. Lancet Oncol. 2012;13(6):607-15. 10.1016/S1470-2045(12)70137-7 22575588

[16] Orrskog S Medin E Tsolova S Semenza JC Causal inference regarding infectious aetiology of chronic conditions: a systematic review. PLoS One. 2013;8(7):e68861. 10.1371/journal.pone.0068861 23935899 PMC3723854

[17] van Lier EA Havelaar AH Nanda A The burden of infectious diseases in Europe: a pilot study. Euro Surveill. 2007;12(12):751. 10.2807/esm.12.12.00751-en 18076860

[18] Jakab Z Why a burden of disease study? Euro Surveill. 2007;12(12):750. 10.2807/esm.12.12.00750-en 18076856

[19] Kretzschmar M Mangen MJ Pinheiro P Jahn B Fèvre EM Longhi S New methodology for estimating the burden of infectious diseases in Europe. PLoS Med. 2012;9(4):e1001205. 10.1371/journal.pmed.1001205 22529750 PMC3328443

[20] Mangen MJ Plass D Havelaar AH Gibbons CL Cassini A Mühlberger N The pathogen- and incidence-based DALY approach: an appropriate [corrected] methodology for estimating the burden of infectious diseases. PLoS One. 2013;8(11):e79740. 10.1371/journal.pone.0079740 24278167 PMC3835936

[21] Murray CJ Lopez AD Global mortality, disability, and the contribution of risk factors: Global Burden of Disease Study. Lancet. 1997;349(9063):1436-42. 10.1016/S0140-6736(96)07495-8 9164317

[22] Murray CJ Quantifying the burden of disease: the technical basis for disability-adjusted life years. Bull World Health Organ. 1994;72(3):429-45. 8062401 PMC2486718

[23] Murray CJ Ezzati M Flaxman AD Lim S Lozano R Michaud C GBD 2010: design, definitions, and metrics. Lancet. 2012;380(9859):2063-6. 10.1016/S0140-6736(12)61899-6 23245602

[24] Haagsma JA Maertens de Noordhout C Polinder S Vos T Havelaar AH Cassini A Assessing disability weights based on the responses of 30,660 people from four European countries. Popul Health Metr. 2015;13(1):10. 10.1186/s12963-015-0042-4 26778920 PMC4715333

[25] European Centre for Disease Prevention and Control (ECDC). BCoDE toolkit. Version 1.2. Stockholm: ECDC; 2015. Available from: https://ecdc.europa.eu/en/toolkit-application-calculate-dalys

[26] The European Parliament and the Council of the European Union. Decision No 2119/98/EC of the European Parliament and of the Council of 24 September 1998 setting up a network for the epidemiological surveillance and control of communicable diseases in the Community. Official Journal of the European Union. Luxembourg: Publications Office of the European Union. 3.10.1998:L 268. Available from: http://eur-lex.europa.eu/LexUriServ/LexUriServ.do?uri=CELEX:31998D2119:EN:HTML

[27] The European Parliament and the Council of the European Union. Regulation (EC) No 851/2004 of the European Parliament and of the Council of 21 April 2004 establishing a European centre for disease prevention and control. Official Journal of the European Union. Luxembourg: Publications Office of the European Union. 30.4.2004:L 142. Available from: http://ecdc.europa.eu/en/aboutus/Key%20Documents/0404_KD_Regulation_establishing_ECDC.pdf

[28] Eurostat. Population on 1 January by age and sex, 2011. Luxembourg: Eurostat. [Accessed 1 May 2016]. Available from: http://appsso.eurostat.ec.europa.eu/nui/show.do?dataset=demo_pjan&lang=en

[29] Gibbons CL Mangen MJ Plass D Havelaar AH Brooke RJ Kramarz P Measuring underreporting and under-ascertainment in infectious disease datasets: a comparison of methods. BMC Public Health. 2014;14(1):147. 10.1186/1471-2458-14-147 24517715 PMC4015559

[30] Hayward AC Fragaszy EB Bermingham A Wang L Copas A Edmunds WJ Comparative community burden and severity of seasonal and pandemic influenza: results of the Flu Watch cohort study. Lancet Respir Med. 2014;2(6):445-54. 10.1016/S2213-2600(14)70034-7 24717637 PMC7164821

[31] Murray CLA. Rethinking DALYs. In: Murray CLA, Lopez AD, editors. The global burden of disease: a comprehensive assessment of mortality and disability from diseases, injuries, and risk factors in 1990 and projected to 2020. Boston: Harvard School of Public Health; 1996. p.1-98.

[32] Mangen MJJ, Kretzschmar MEE. Impact of time trends and the time span of data collection on disease burden estimates of infectious diseases. In: Mangen MJM, Plass D, Ktretzschmar MEE, editors. Estimating the current and future burden of communicable diseases in the European Union and EEA/EFTA. Bilthoven: National Institute for Public Health and the Environment; 2014. p. 66-71.

[33] Devleesschauwer B Haagsma JA Angulo FJ Bellinger DC Cole D Döpfer D Methodological Framework for World Health Organization Estimates of the Global Burden of Foodborne Disease. PLoS One. 2015;10(12):e0142498. 10.1371/journal.pone.0142498 26633883 PMC4668830

[34] Muscat M Marinova L Mankertz A Gatcheva N Mihneva Z Santibanez S The measles outbreak in Bulgaria, 2009-2011: An epidemiological assessment and lessons learnt. Euro Surveill. 2016;21(9):30152. 10.2807/1560-7917.ES.2016.21.9.30152 26967661

[35] European Centre for Disease Prevention and Control (ECDC). Revised estimates of deaths associated with seasonal influenza in the US. ECDC Comment. Stockholm: ECDC; 24 Oct 2010. Available from: https://ecdc.europa.eu/en/news-events/revised-estimates-deaths-associated-seasonal-influenza-us

[36] van den Wijngaard CC van Asten L Koopmans MP van Pelt W Nagelkerke NJ Wielders CC Comparing pandemic to seasonal influenza mortality: moderate impact overall but high mortality in young children. PLoS One. 2012;7(2):e31197. 10.1371/journal.pone.0031197 22319616 PMC3272034

[37] National Institute for Public Health and the Environment (RIVM). State of Infectious Diseases in the Netherlands, 2013. Bilthoven: RIVM; 2014. Available from: http://www.rivm.nl/bibliotheek/rapporten/150205001.pdf

[38] National Institute for Public Health and the Environment (RIVM). State of Infectious diseases in the Netherlands, 2015. Bilthoven: RIVM; 2016. Available from: http://www.rivm.nl/bibliotheek/rapporten/2016-0069.pdf

[39] Council of the European Union. Council Recommendation of 22 December 2009 on seasonal influenza vaccination (Text with EEA relevance). Official Journal of the European Union. Luxembourg: Publications Office of the European Union. 29.12.2009: L 348/71. Available from: http://eur-lex.europa.eu/LexUriServ/LexUriServ.do?uri=OJ:L:2009:348:0071:0072:EN:PDF

[40] European Centre for Disease Prevention and Control (ECDC). Seasonal influenza vaccination in Europe. Vaccination recommendations and coverage rates in the EU Member States for eight influenza seasons: 2007–2008 to 2014–2015. Stockholm: ECDC, Jul 2017. Available from: https://ecdc.europa.eu/sites/portal/files/documents/influenza-vaccination-2007%E2%80%932008-to-2014%E2%80%932015.pdf

[41] Eurostat. Causes of death - standardised death rate by residence. Luxembourg: Eurostat. [Accessed 1 Aug 2016]. Available from: http://ec.europa.eu/eurostat/data/database

[42] European Centre for Disease Prevention and Control (ECDC)/World Health Organization Regional Office for Europe (WHO/Europe). HIV/AIDS surveillance in Europe 2014. Stockholm: ECDC; 2015. Available from: https://ecdc.europa.eu/sites/portal/files/media/en/publications/Publications/hiv-aids-surveillance-in-Europe-2014.pdf

[43] Navarro-Torné A Dias JG Hruba F Lopalco PL Pastore-Celentano L Gauci AJ Invasive Pneumococcal Disease Study Group Risk factors for death from invasive pneumococcal disease, Europe, 2010. Emerg Infect Dis. 2015;21(3):417-25. 10.3201/eid2103.140634 25693604 PMC4344260

[44] van Deursen AM van Mens SP Sanders EA Vlaminckx BJ de Melker HE Schouls LM Invasive pneumococcal disease and 7-valent pneumococcal conjugate vaccine, the Netherlands. Emerg Infect Dis. 2012;18(11):1729-37. 10.3201/eid1811.120329 23092683 PMC3559145

[45] European Centre for Disease Prevention and Control (ECDC). Surveillance of invasive bacterial diseases in Europe, 2012. Stockholm: ECDC; 2015. Available from: https://ecdc.europa.eu/sites/portal/files/media/en/publications/Publications/Surveillance%20of%20IBD%20in%20Europe%202012.pdf

[46] Cassini A Plachouras D Eckmanns T Abu Sin M Blank H-P Ducomble T Burden of Six Healthcare-Associated Infections on European Population Health: Estimating Incidence-Based Disability-Adjusted Life Years through a Population Prevalence-Based Modelling Study. PLoS Med. 2016;13(10):e1002150. 10.1371/journal.pmed.1002150 27755545 PMC5068791

[47] Mayhall CG, editor. Hospital Epidemiology and Infection Control. 4th ed. Philadelphia: Lippincott Williams & Wilkins; 2012.

[48] Jarvis WR, editor. Bennett & Brachman's Hospital Infections. 6th ed. Philadelphia: Lippincott Williams & Wilkins; 2014.

[49] Kirk MD Pires SM Black RE Caipo M Crump JA Devleesschauwer B World Health Organization Estimates of the Global and Regional Disease Burden of 22 Foodborne Bacterial, Protozoal, and Viral Diseases, 2010: A Data Synthesis. PLoS Med. 2015;12(12):e1001921. 10.1371/journal.pmed.1001921 26633831 PMC4668831

[50] Hanquet G Perrocheau A Kissling E Bruhl DL Tarragó D Stuart J Surveillance of invasive pneumococcal disease in 30 EU countries: Towards a European system? Vaccine. 2010;28(23):3920-8. 10.1016/j.vaccine.2010.03.069 20394721

[51] Wichmann O Hellenbrand W Sagebiel D Santibanez S Ahlemeyer G Vogt G Large measles outbreak at a German public school, 2006. Pediatr Infect Dis J. 2007;26(9):782-6. 10.1097/INF.0b013e318060aca1 17721371

[52] Stein CE Birmingham M Kurian M Duclos P Strebel P The global burden of measles in the year 2000--a model that uses country-specific indicators. J Infect Dis. 2003;187(s1) Suppl 1;S8-14. 10.1086/368114 12721886

[53] Wolfson LJ Strebel PM Gacic-Dobo M Hoekstra EJ McFarland JW Hersh BS Has the 2005 measles mortality reduction goal been achieved? A natural history modelling study. Lancet. 2007;369(9557):191-200. 10.1016/S0140-6736(07)60107-X 17240285

[54] Haagsma JA Polinder S Cassini A Colzani E Havelaar AH Review of disability weight studies: comparison of methodological choices and values. Popul Health Metr. 2014;12(1):20. 10.1186/s12963-014-0020-2 26019690 PMC4445691

[55] Colzani E Cassini A Lewandowski D Mangen MJ Plass D McDonald SA A Software Tool for Estimation of Burden of Infectious Diseases in Europe Using Incidence-Based Disability Adjusted Life Years. PLoS One. 2017;12(1):e0170662. 10.1371/journal.pone.0170662 28107447 PMC5249178

[56] EFSA Panel on Biological Hazards Scientific Opinion on the development of a risk ranking toolbox for the EFSA BIOHAZ Panel. EFSA J. 2015;13(1):3939 10.2903/j.efsa.2015.3939

[57] Fafangel M Cassini A Colzani E Klavs I Grgič Vitek M Učakar V Estimating the annual burden of tick-borne encephalitis to inform vaccination policy, Slovenia, 2009 to 2013. Euro Surveill. 2017;22(16):30509. 10.2807/1560-7917.ES.2017.22.16.30509 28449731 PMC5404479

[58] Cassini A Colzani E Kramarz P Kretzschmar M Takkinen J Impact of food and water-borne diseases on European population health. Current Opinion in Food Science. 2016;12:21-9. 10.1016/j.cofs.2016.06.002

[59] European Centre for Disease Prevention and Control (ECDC). Surveillance Atlas of Infectious Diseases. Stockholm: ECDC. [Accessed 1 Aug 2016]. Available from: https://ecdc.europa.eu/en/surveillance-atlas-infectious-diseases

[60] Cassini AMK, Emborg HD, Simonsen J, Teunis P, van Pelt W, Takkinen J. Estimation of incidence of symptomatic salmonella and campylobacter infections based on sero-incidence in 13 European countries. European Scientific Conference on Applied Infectious Disease Epidemiology (ESCAIDE); Stockholm, Sweden: European Centre for Disease Prevention and Control (ECDC); 2015.

[61] European Centre for Disease Prevention and Control (ECDC). Annual Epidemiological Reports. Stockholm: ECDC. [Accessed 1 Apr 2016]. Available from: https://ecdc.europa.eu/en/annual-epidemiological-reports

[62] European Centre for Disease Prevention and Control (ECDC)/World Health Organization Regional Office for Europe (WHO/Europe). Tuberculosis surveillance and monitoring in Europe 2016. Stockholm: ECDC; 2016. Available from: https://ecdc.europa.eu/sites/portal/files/media/en/publications/Publications/ecdc-tuberculosis-surveillance-monitoring-Europe-2016.pdf

